# Acid-active proteases to optimize dietary protein digestibility: a step towards sustainable nutrition

**DOI:** 10.3389/fnut.2024.1291685

**Published:** 2024-02-08

**Authors:** Wai Shun Mak, Chloe P. Jones, Kevin E. McBride, Emily A. P. Fritz, Julie Hirsch, J. Bruce German, Justin B. Siegel

**Affiliations:** ^1^Department of Chemistry, University of California, Davis, Davis, CA, United States; ^2^Digestiva, Inc., Davis, CA, United States; ^3^Empowered Science, LLC, South Barrington, IL, United States; ^4^Department of Food Science and Technology, University of California, Davis, Davis, CA, United States; ^5^Genome Center, University of California, Davis, Davis, CA, United States; ^6^Department of Biochemistry and Molecular Medicine, University of California, Davis, Davis, CA, United States

**Keywords:** sustainability, *in vitro* digestion, acid protease, sustainable nutrition, nutrition security, protein digestibility

## Abstract

**Introduction:**

Historically, prioritizing abundant food production often resulted in overlooking nutrient quality and bioavailability, however, environmental concerns have now propelled sustainable nutrition and health efficacy to the forefront of global attention. In fact, increasing demand for protein is the major challenge facing the food system in the 21st century with an estimation that 70% more food is needed by 2050. This shift has spurred interest in plant-based proteins for their sustainability and health benefits, but most alternative sources of protein are poorly digestible. There are two approaches to solve digestibility: improve the digestibility of food proteins or improve the digestive capacity of consumers. Enhancing nutrient digestibility and bioavailability across diverse protein sources is crucial, with proteases presenting a promising avenue. Research, inspired by the proteases of human breast milk, has demonstrated that exogenous microbial proteases can activate within the human digestive tract and substantially increase the digestion of targeted proteins that are otherwise difficult to fully digest.

**Methods:**

Here, we introduce the use of an acid-active family of bacterial proteases (S53) to improve the digestibility and nutritional quality of a variety of protein sources, evaluated using the INFOGEST 2.0 protocol.

**Results:**

Results from *in vitro* digestibility indicate that the most effective protease in the S53 family substantially improves the digestibility of an array of animal and plant-derived proteins—soy, pea, chickpea, rice, casein, and whey. On average, this protease elevated protein digestibility by 115% during the gastric phase and by 15% in the intestinal phase, based on the degree of hydrolysis.

**Discussion:**

The widespread adoption of these proteases has the potential to enhance nutritional value and contribute to food security and sustainability. This approach would complement ongoing efforts to improve proteins in the food supply, increase the quality of more sustainable protein sources and aid in the nourishment of patients with clinically compromised, fragile intestines and individuals like older adults and high-performance athletes who have elevated protein needs.

## 1 Introduction

Achieving climate change goals to avert global catastrophe will require a literal revolution in the agriculture and food sector. Everything from agricultural efficiency to food waste must play a part including consumer preferences, as one-third of shoppers consider eco-friendly options a top priority (1). This shift in consumer priorities has been fueled by rapid environmental changes, population growth, and an increasing awareness of the health and ecological impacts of dietary choices (2). Plant-based diets continue to be a centerpiece of sustainable solutions that can reduce the environmental impact, but there is concern that this transition may compromise nutritional quality and health and lead to the creation of a sustainability paradox (3). At the center of the problem is protein.

Consuming adequate protein is a key nutritional consideration to achieve health on any diet. This is especially true for populations like adolescents for growth and development, physically active individuals for optimal physical performance, and older adults to reduce the risk of conditions like sarcopenia or sarcopenic obesity (4–7). Further, the combination of plant-based or vegan diets with even small amounts of physical activity can compound the net nutrition issue, making adequate protein intakes difficult to achieve (8, 9). A chief concern is that many plant-based protein sources lag behind animal proteins due to limitations in essential amino acids and digestibility (3, 10, 11). This issue is further exaggerated by the nutrikinetics of protein digestion and absorption. Although protein quality for certain animal and plant sources might be high, consumption of certain proteins and the influence of other nutrients can result in a slower rise in post-prandial amino acidosis which has implications for protein metabolism (12). These issues can all be compounded by a greater focus on a minimally processed, whole foods which have been shown to further reduce the digestion and absorption of proteins from animal, plant and microbial sources (13–15).

In this context, protease technology emerges as a promising solution to maximize the health benefits of plant-based protein and support sustainable choices without nutritional compromise since protein complementation alone will not solve the limitation in essential amino acids. Proteases within the human gastrointestinal tract have undergone evolutionary adaptations that have resulted in increasingly general activity, possessing broad substrate specificity. This generalist characteristic allows humans the ability to hydrolyze a diverse array of food proteins during digestion. However, this advantage is counterbalanced by a compromise in enzymatic specificity and catalytic efficiency, leading to the suboptimal digestion and utilization of nutrients and concomitant challenges within the digestive system. Of particular note, is the challenge presented by plant-derived proteins, which often exhibit complex structures and contain anti-nutritional factors, limiting protein digestion and utilization. This phenomenon is of heightened concern in geriatric populations, where the intrinsic decline in digestive function and protein metabolism with advancing age exacerbates the challenge (16, 17).

The structural diversity among dietary proteins affecting their digestibility is resolved biologically by a paralleled variability in proteases, each differing in activity and substrate specificity. In practice, this biological complexity necessitates the precise pairing of a protease with its corresponding substrate (18). Matching biological enzyme diversity to biological protein targets was too complex to be accessible by traditional food practices but is now a process that can be guided by genomic mining (19). Phylogenetic databases have uncovered enzymes specifically tailored for targeted reactions. Nature’s evolutionary process has given rise to a wide array of enzymes, enabling different organisms to thrive in diverse environments, and ensuring that no protein source remains non-biodegradable. This inherent adaptability is evidenced in large enzyme families, where research has revealed unexpected substrate specificities and novel functions, promising innovative applications (20). As the global trend towards plant-based diets gathers momentum, the strategic exploration of nature’s vast protease reservoir becomes vital. By harnessing this evolutionary legacy, protein digestion can be optimized to support the transformation of our global food system, aligning with broad and potentially disruptive dietary shifts.

The objective of this paper is to provide an overview of discovery and screening of proteases from the S53 family and the *in vitro* effectiveness of the leading candidate family member (P24) in improving protein digestibility using the INFOGEST 2.0 *in vitro* digestion model (21). The S53 protease family, also known as sedolisins or serine-carboxyl peptidases, was originally identified in the early 1980s by Murao, Oda, and their colleagues (22). They discovered a group of endopeptidases with acidic pH optima that were noted for their resistance to inhibition by pepstatin, making them prime targets for nutritional applications. This novel family of proteases exhibit activity under acidic conditions, making it an excellent candidate for application in enhancing *in vivo* gastric digestion (23). There are over 13,000 different proteases in the S53 family and 12 of them are characterized in detail here with particular attention to the most promising candidates for improving the bioavailability of a wide range of plant and animal proteins.

In this work, we present the screening results of various food proteins combined with S53 proteases, establishing a foundational understanding of their broad-spectrum efficacy. Subsequently, in our second dataset we delve into a more detailed assessment of a promising S53 protease, P24, using the INFOGEST 2.0 simulated digestion protocol. The INFOGEST 2.0 results offer a quantitative measure of the impact P24 has on specific protein sources. This two-tiered approach allows for a comprehensive evaluation of S53 proteases’ and the potential of P24 in enhancing the nutritional quality of diverse proteins. The application of acid activated S53 protease family members holds significant potential for global impact. These enzymes could unlock readily available sources of plant-based proteins that are becoming increasingly prominent in the dietary patterns of a wide range of consumers. By improving the digestibility and bioavailability of such proteins, protease technologies can contribute to a sustainable future where nutrient dense food alternatives are readily available all the while contributing to our planet’s sustainability goals (1, 3).

## 2 Materials and methods

### 2.1 Protease expression and purification

The enzymes (proteases) for all experiments were synthesized in the Digestiva, Inc. labs. Each protease is labeled by their protein accession number (unique identifier given to a DNA or protein sequence record to allow for tracking of different versions). Details of enzyme sequences and structure can be found in UniprotKB database. All genes tested in this work were codon optimized for *Escherichia coli* and synthesized by Thermo Fisher GeneArt synthesis technology into our expression plasmid pET-29 b (+) such that each coding region included a C-terminal 6xHis tag. The sequence verified plasmids were transformed into the BLR strain of *E. coli* for protein expression. The expression procedures began with the inoculation of transformed *E. coli* from glycerol stocks in to 0.5–5 liters, depending on the protein expression level of each gene, (Supplementary Table S2) of terrific broth (1 mM of MgSO4, 0.4% glucose (w/v) and 50 μg/mL kanamycin) at 37°C for 24 h in 1-liter baffled-flasks with 500 mL of media in each flask shaking at 300 rpm. These cultures were then spun down at 4816 g for 10 min and resuspended in induction media ((II) ferrous sulfate, 1 mM of MgSO4, 1× 5,052 (0.5% glycerol, 0.05% glucose, 0.2% alpha-lactose) solution, 1x NPS (1 M potassium phosphate monobasic, 1 M sodium phosphate dibasic, 0.5 M ammonium sulfate) solution and 50 mg/mL kanamycin) and shaken at 300 rpm for 33–35 h at 18°C. At the end of induction, these cultures were spun down at 4816 g for 15 min and resuspended in 25 mL of lysis buffer (50 mM HEPES pH 7.5, 200 mM NaCl, 1 mM TCEP, 2 mM imidazole, 1 mM PMSF). The resuspended cultures were then submerged in water-ice bath and lyzed with sonication using Fisher Scientific^™^ Model 705 Sonic Dismembrator for 30 s on and 30 s off interval at an amplitude of 35 with a total sonication-on time of 2 min.

The lysed cultures were then clarified by centrifugation at 4816 g for 1 h at 4°C. Supernatant from clarified cultures were loaded on columns with 500 mL of cobalt resin to pull down the His-tagged proteins. The resin bed was subsequently washed with 10 mL of wash buffer (50 mM HEPES pH 7.5, 200 mM NaCl, 1 mM TCEP, 2 mM imidazole) 3 times and eluted with 500 mL of elution buffer (50 mM HEPES pH 7.5, 200 mM NaCl, 1 mM TCEP, 200 mM imidazole). The purified proteins were immediately buffer exchange into storage buffer (50 mM HEPES pH 7.5, 200 mM NaCl, 1 mM TCEP) and assayed within 24 h of purification.

Protein concentrations were determined using an Epoch spectrophotometer (Biotek) at 280 nm using their calculated extinction coefficients with the ExPASy ProtParam Tool. All other buffers and salts were purchased from Fisher Scientific, unless otherwise specified.

### 2.2 SDS-PAGE screening of protease activity in protein substrates

#### 2.2.1 Protein substrates

Protease digestive activity for twelve (12) enzymes was determined for the digestion of thirty-one (31) plants (grains, legumes, nuts, seeds, protein extracts) and animal protein substrates. All protein sources were provided directly by the manufacturers, ingredient broker, or purchased locally at retailers. Details of the proteins can be found in the Supplementary Table S1.

#### 2.2.2 Enzyme activity method using SDS-PAGE electrophoresis and densitometry analysis

Digestion assays were conducted by incubating 2 μM of each respective enzyme with individual protein sources at 37°C for a 12-h duration, utilizing a reaction buffer of pH 4.5 composed of 100 mM acetate and 100 mM NaCl. Following the incubation, samples were centrifuged at 4,700 rpm for 10 min to sediment any undigested material. The supernatant was then mixed with 1X Laemmli buffer and heated at 70°C for 10 min to denature proteins and terminate enzymatic activity. These prepared samples were loaded onto a 12% polyacrylamide gel and subjected to electrophoresis. Upon completion, the gel was stained with Coomassie blue to visualize protein bands.

To quantify proteolytic activities, an in-house analytical method was employed, which involved converting the gel image to grayscale for better contrast and densitometry analysis. Thresholding techniques were applied to the grayscale image to distinguish protein bands from background noise. Contour mapping was then used to isolate individual lanes on the gel based on preset dimensional parameters. Each lane’s contour was analyzed for its total pixel density, which represented the sum of grayscale values within the bounded region. A darker background lane was used for background subtraction, and the total density of a control lane served as a reference point for calculating the percent digestion for each lane, thereby allowing for the nuanced quantification of proteolytic activity.

### 2.3 *In vitro* static digestion with INFOGEST protocol

#### 2.3.1 Protein substrates

*In vitro* digestion was determined for six (6) plant and animal protein substrates (Table 1). All protein sources were provided directly by the manufacturers, ingredient broker, or purchased locally at retailers.

**Table 1:** INFOGEST protein information.

Protein raw material	Product brand name	Manufacturer	Percent protein by dry weight
Soy protein isolate	SUPRO XT^®^ 219D	Solae^™^	90% Min
Pea protein isolate	Puris 870	Puris	80% Min
Chickpea protein concentrate	Artesa^™^ 100-P-1	Tate & Lyle	60% Min
Rice protein isolate	VitaPro^™^ RI 80	Austrade	80% Min
Casein	Naked Casein	Naked	80% Min
Whey	SureProtein^™^ whey protein isolate 8,855	Fonterra	88% Min

#### 2.3.2 INFOGEST 2.0 protocol method

*In vitro* digestion was conducted in alignment with the INFOGEST 2.0 consensus protocol (21). Chemicals and enzymes were purchased from Sigma-Aldrich unless stated otherwise. Simulated salivary (SSF), gastric (SGF) and intestinal fluid (SIF) digestive solutions were used. Enzyme activities and bile concentrations were determined using protocols from the Supplementary methods section of the INFOGEST 2.0 protocol. To ensure the precise addition of appropriate enzyme activities detailed in the INFOGEST 2.0 protocol, both trypsin and chymotrypsin activity levels were determined for the pancreatin for normalization. The enzymes used were porcine pepsin (cat. no. P6887, lot no. SLCM5196), porcine trypsin (cat. no. T0303, lot no. SLBX8983), and porcine pancreatin (cat. no. P7545, lot no. SLCF4576). For bile, bovine bile (cat. no. B3883, lot no. SLCG9142) was used. 2.5 g of protein extract powder (dry basis) (Table 1), were combined with 12.5 mL of 1.25X SSF for the salivary phase, and 25 mL of 1.25X SGF for the gastric phase. The pH was adjusted to 3.0 via titration with 1 M HCl, and the volume was augmented to 50 mL with MilliQ water, normalizing the final protein concentration to 50 mg/mL.

Salivary digestion with simulated salivary fluid (SSF) took place for proper dilution of proteins. For the initiation of the gastric phase digestion, 4 mL of the substrate protein solution was dispensed into five 50-mL conical tubes. Tubes 1–3 were supplemented with 0.5 mL of pepsin and 0.5 mL of P24 stock, while tube 4 received 0.5 mL of pepsin and 0.5 mL of 0.2X Phosphate buffer saline (PBS). Tube 5, serving as a background control, was given 0.5 mL of 10 mM Tris, 150 mM NaCl pH 6.4 buffer, and 0.5 mL of 0.2X PBS. After enzyme addition, the tubes were agitated at 300 rpm in a 37°C MaxQ^™^ 4,000 benchtop orbital shaker for 2 h. The reaction was quenched with 300 μL of 1 M NaOH, followed by the addition of 2.3 mL of a combined bile salt and SIF solution to attain a final concentration of 10 mM bile salts. The pH was carefully adjusted to 7.00 ± 0.05 using 1 M NaOH. A 2.9 mL sample of digesta was extracted for a gastric timepoint, treated with 1 mL of water, and 1.5 mL of the resultant solution was subjected to heating at 85°C for 15 min to simulate intestinal sample quenching.

For the intestinal phase, 1.6 mL of pancreatin and trypsin solutions were introduced into the first four enzymatic conical tubes, adjusting the final chymotrypsin and trypsin activities to 25 U/mL and 100 U/mL, respectively. The background tube received 1.6 mL of water decreased to pH 3 with 6 M HCl, resulting in a uniform final volume of 5 mL per tube. After a 10-min shaking interval, 1.5 mL of digesta was extracted and quenched at 85°C for 15 min. This process was repeated after a further 2-h shaking period. The three timepoints—gastric, 10-min intestinal, and 2-h intestinal—were then centrifuged, and the supernatants were collected.

#### 2.3.3 Determination of degree of hydrolysis in INFOGEST 2.0 digesta

The analysis of amines was conducted using a fluorescamine assay. Initially, the digesta supernatant was diluted 100-fold in 1X PBS and then allocated in triplicate to a black opaque 96-well plate (Corning, cat. no. 3960). To each well, 70 μL of 50 mM sodium phosphate buffer at pH 7 was added, followed by the addition of 20 μL of 0.5 mg/mL Fluorescamine (cat no. F9015, batch #0000128324) in acetonitrile, commencing the reaction. Immediate mixing and a subsequent 5-min incubation facilitated the reaction, after which the fluorescence was quantified at Ex/Em 390 nm/475 nm using a Spectramax microplate reader. A peptide standard curve was established for each assay using a peptide standard (ThermoScientific, cat. no. 23295). This enabled the calculation of the percent degree of hydrolysis through this predefined equation for each digestion sample.


$$\%DH=\frac{aa_{\mathrm{digesta}}\left(\mathrm{mol}\right)}{aa_{\mathrm{protein}\;\mathrm{source}}\left(g\right)\times \frac{1}{110}\mathrm{mol}/g}\times 100.$$

During this process, the amino acid weight in the diluent was kept constant at 0.02 g/mL. The standard error was then ascertained by averaging the values derived from three consecutive days, with the data from the three P24 tubes amalgamated to provide a single representative value for each day.

Statistical analysis was conducted using JMP (JMP^®^, Version 17. SAS Institute Inc., Cary, NC, 1989–2023.) where comparisons of percent degree of hydrolysis were conducted for each source of protein between simulated digestive fluids (SDF) and simulated digestive fluids plus P24 (SDF + P24) at the end of gastric phase (GP, 120 min) and at 10 min into the intestinal phase (IP 10) and 120 min into the intestinal phase (IP 120). Tests for normality (Shapiro–Wilk) and homoscedasticity (Bartlett’s Test) confirmed the data met appropriate assumptions for a comparison of means via paired Student’s t-tests using Fisher’s least significant difference method. Significance was reported at *p* < 0.05.

## 3 Results

**Figure 1:**
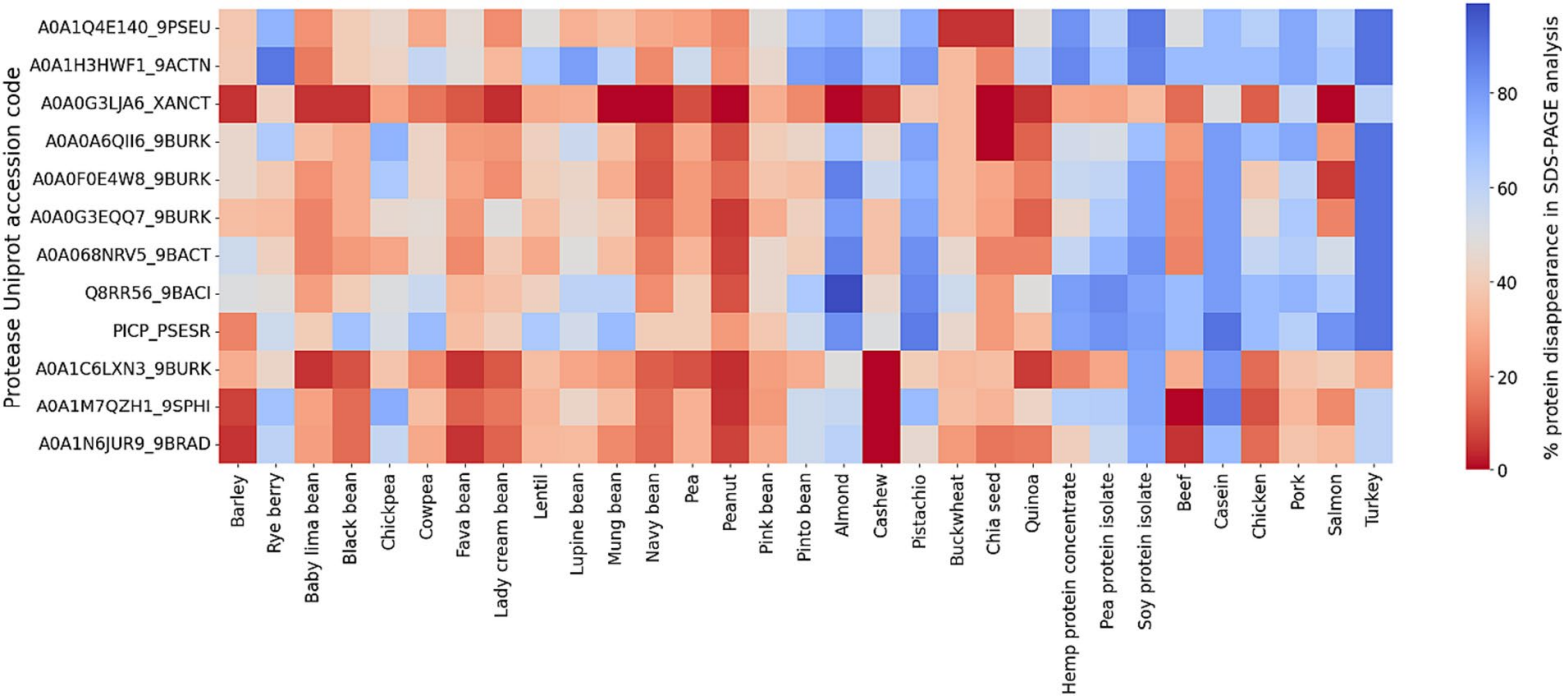
Heatmap representation of protein bands disappearance in SDS-PAGE screening for 12 bacterial proteases (S53 family) against 31 protein sources. The color coding indicates the efficiency of protein digestion, with blue representing disappearance (i.e., protein degradation) on an SDS-PAGE gel and red representing no digestion.

In our SDS-PAGE screening of protease activity, the data are presented as the disappearance of percent protein bands density from the gel of all protein substrates relative to a control without protease. An empty lane is defined as 100% digestion in this qualitative screening assay. An examination of the average degree of hydrolysis across protein classification and type, for all the proteases, reveals notable variation (Figure 1). The greatest percent of protein bands disappearance occurred in proteins from commercially available protein isolates (pea, soy, hemp) with an average of 59%, indicating that the refined nature of the isolated and concentrated proteins render them more susceptible to enzymatic breakdown (e.g., compare pea to pea protein isolate). Animal-based proteins follow closely with an average protein disappearance of 57%, followed by legumes (34%), seeds (39%), and finally, grains with the lowest average disappearance (31%). These values reflect the heterogeneous nature of protein structures and the consequential influence on digestibility (all SDS-PAGE results can be found in Supplementary Figure S1).

At the enzyme level, it is important to note that there is a wide difference in digestive activity of the different proteases. The enzymes which demonstrate the greatest efficacy include Q8RR56_9BACI with the highest digestibility in three categories: protein extracts (59%), animal-based proteins (57%), and nuts and seeds (39%). A0A068NRV5_9BACT performs well in grains and seeds and PICP_PSESR efficient at digesting legumes.

Conversely, A0A0G3LJA6_XANCT records the lowest digestibility on average, and A0A1N6JUR9_9BRAD is least effective in animal-based proteins among all proteases tested in this work. The subpar performance of these enzymes indicates potential limitations in their digestive capacities. These results confirm the need for specificity of enzyme action on different protein sources and structures, affirming the need for precise proteases applications.

In a broader context, the data delineate distinct digestibility patterns between animal and plant-based proteins. Animal sources, are overall better digested across the array of proteases versus the plant proteins. Overall, turkey, an animal derivative, is the most digestible protein across all proteases. Conversely, peanut, a plant protein (legume), emerges as the least digestible.

### 3.1 INFOGEST 2.0 protocol and detailed examination of protease Q8RR56_9BACI

The INFOGEST 2.0 protocol simulates both the gastric and intestinal phases of digestion, and provides a more reliable predictor of human digestibility. Protease enzyme Q8RR56_9BACI (henceforth designated as P24 for succinctness) was further explored using the INFOGEST 2.0 protocol based on its strong digestive performance across multiple protein categories during protease activity screening.

The impact of P24 on protein digestibility as part of the simulated digestive fluid (SDF + P24) was compared to simulated digestive fluid (SDF) alone during the gastric phase (GP), 10 min into the intestinal phase (IP 10), and 120 min into the intestinal phase (IP 120). Overall, the addition of P24 to the gastric phase (GP, SDF + P24) significantly outperforms the protein digestibility when compared to SDF alone. During the gastric phase (GP), P24 improved digestibility for all sources of protein with SDF + P24 resulting in an average of 7.18% degree hydrolysis and SDF alone at 3.32% (*p < 0.001*). During both the early (IP 10) and late intestinal phase (IP 120) the significant difference between SDF + P24 and SDF continued with 12.8% compared to 10.98% at IP10 (*p = 0.0002*) and 13.98% compared to 12.35%, respectively (*p = 0.006*). In addition to the benefits of P24 overall, there were statistically significant improvements in digestibility for each individual protein source during GP, and increased digestibility during the IP 10 and IP 120 for all protein sources except for whey protein shown in Table 2 (a graphical representation of the comparison of digestibility by protein source can be found in the Supplementary Figure S2).

**Table 2:** Percent degree of hydrolysis by protein source measured by INFOGEST 2.0 with and without P24.

	No P24	P24	Std error difference	*p*-value
**Total**				
Gastric	3.32%	7.18%	0.23%	<0.0001
Intestinal 10 min	10.98%	12.80%	0.14%	0.0002
Intestinal 120 min	12.35%	13.98%	0.16%	0.0006
**Soy**				
Gastric	3.60%	7.80%	0.34%	0.0002
Intestinal 10 min	11.70%	14.37%	0.74%	0.0231
Intestinal 120 min	13.28%	14.68%	0.47%	0.0416
**Pea**				
Gastric	3.21%	7.25%	0.21%	<0.0001
Intestinal 10 min	8.40%	12.18%	0.36%	0.0005
Intestinal 120 min	9.12%	12.32%	0.24%	0.0002
**Chickpea**				
Gastric	3.32%	6.23%	0.23%	0.0002
Intestinal 10 min	7.16%	8.86%	0.25%	0.0024
Intestinal 120 min	9.09%	11.35%	0.64%	0.0252
**Rice**				
Gastric	2.52%	6.09%	0.53%	0.0006
Intestinal 10 min	8.92%	9.60%	0.13%	0.002
Intestinal 120 min	10.64%	12.67%	0.28%	0.0004
**Casein**				
Gastric	4.61%	11.12%	1.40%	0.0098
Intestinal 10 min	14.56%	15.91%	0.24%	0.0053
Intestinal 120 min	15.71%	16.37%	0.19%	0.0255
**Whey**				
Gastric	2.82%	5.01%	0.29%	0.0003
Intestinal 10 min	15.05%	15.77%	0.44%	0.1538
Intestinal 120 min	16.20%	16.54%	0.36%	0.3767

## 4 Discussion

Data from this comprehensive enzymatic evaluation support the benefits of P24’s activity toward enhancing protein digestion and bioavailability, thereby facilitating improved nutrient absorption. As the world shifts towards prioritizing protein nutrition, the findings on P24’s capabilities present a promising candidate for bolstering sustainable nutrition solutions through the application of acid-activated proteases. Additionally, the SDS-PAGE screening of digestive activity for the S53 family of proteases indicate the broad possibilities for the application of proteases in a wide array of sustainable protein sources with greater substrate specificity.

The sustainability and health implications of dietary choices have been at the forefront of global concerns. Plant-based diets are increasingly viewed as an environmentally friendly alternative to omnivorous diets high in animal-derived proteins with the promise of reduced environmental impact and the potential for additional health benefits (24). However, challenges related to the digestibility, amino acid profile, and bioavailability of plant proteins and plant-based foods need to be addressed to ensure they can optimize health and nutritional needs. Although recommendations for improving the nutritional benefits of plant based protein have been proposed (25), there is a need for the novel solution that the S53 family of acid active proteases can provide as evidenced by the improvement in hydrolysis achieved in both the gastric and intestinal phases of INFOGEST 2.0 for a range of plant and animal proteins. Additionally, the results of qualitative screening indicate future potential for other members of the S53 protease family to improve protein digestion with targeted activity for specific protein sources.

Versions of the INFOGEST static *in vitro* digestion method have been widely used to assess the digestibility of different sources of protein with recent attention being paid to plant-based proteins and adjustments mimicking the simulated digestive conditions of older adult (21, 26, 27). The INFOGEST protocol has also been used to assess the impact of supplemental digestive enzymes with positive results on percent degree of protein hydrolysis (28, 29), however, this is the first study of its kind to evaluate the S53 family of acid-active proteases. Although the findings will need to be replicated in human studies to support the potential benefits, data presented herein follow the recommended first steps for confirming efficacy of P24 with a focus on more sustainable plant-based proteins which have lower digestibility due to antinutritional factors, complex matrices, and processing effects (3, 24, 30). The implications of improving the gastric digestion of plant protein have health implications for a wide range of populations with greater protein needs as greater adoption of plant-based diets continue.

One of the most relevant applications of P24 is improving the anabolic potential of plant-based protein. The type of protein and its source influences amino acid profile and the rate of absorption, thereby impacting muscle protein synthesis (17, 25). Since plant-based proteins are generally associated with lower protein quality with slower post-prandial absorption rates compared to their animal-based counterparts (31), the introduction of acid-activated proteases, especially from the S53 group, would enhance digestibility and absorption kinetics. This nutrikinetic effect would increase the anabolic potential of plant-based proteins to stimulate muscle protein synthesis with an equivalent amount of previously ineffective proteins (13, 17, 25, 32). This phenomenon is believed to be achieved through the more efficient delivery of essential amino acids to peripheral tissues as opposed to first-pass extraction by the gut. Although recent research suggests that plant-based diets may have equivalent impact on muscle protein synthesis to omnivorous diets matched for total protein at or above 1.6 g/kg/day, observational data on total protein intake suggests that achieving the targeted amounts might be difficult for people consuming a plant-based diet (8, 33). Future research of P24 and members of the S53 family will focus on stimulating muscle protein synthesis. In addition to benefiting athletes and plant-based sports nutrition consumers, the potential implications of improving muscle health are of greatest importance to older adults who lose 3–8% of their muscle mass per year after age 30 with acceleration of this loss happening after the age of 60 (34).

Compounding the effects of age-related muscle losses, older adults also face unique nutritional challenges. With physiological changes like reduced production of stomach acid and altered nutrient requirements (35), the elderly are at an elevated risk of malnutrition (36). Since the pepsinogen is activated to pepsin at an optimal pH 1.8 in gastric digestion (37) and P24 is fully activated from pH 2.5 to 4.5, improved post-prandial protein kinetics are likely. Additionally, it is common for older adults to experience a decrease in appetite and energy intake further exacerbating concerns (38). Here, the role of acid-activated proteases becomes even more clear. By enhancing protein digestion, these S53 enzymes can aid in reducing the risk of protein-energy malnutrition in the elderly, a demographic with heightened protein requirements and decreasing total energy intake (5, 34).

The shift towards a plant-based diet offers health benefits, such as reduced risks of chronic diseases (35) and a significant increase in complex carbohydrate intakes that are associated with improved gut health by with improved gut health by stimulating metabolism of the gut microbiota (39). Many of the gut health benefits of a plant-based diet have been associated with nutrients like vitamins, minerals, dietary fiber, and bioactive compounds like polyphenols (40). Nonetheless, proteins are a more complicated, even paradoxical issue. Two consequences of incomplete protein digestion emerge: undigested peptides can affect microbiome metabolism in a net deleterious direction (41), but specific bioactive peptides target intestinal functions in a beneficial direction (42–44). Precision nutrition will be critical for the future of alternative proteins to minimize the deleterious effects and maximize the benefits.

As consumer awareness of the importance of adequate protein intake increases in conjunction with the shift toward sustainable, plant-based diets, more can be done to make the protein quality of plant foods more transparent. The use of the Protein Digestibility–Corrected Amino Acid Score (PDCAAS) as a measure of protein quality (45, 46) has limitations and is minimally visible to consumers on nutrition fact panels in the United States. Further and more recently, PDCAAS has been improved upon by the WHO/FAO/UNU with prioritization of the Digestible Indispensable Amino Acid Score (DIAAS) which underscores the importance of both amino acid profile and digestibility in determining the nutritional value of proteins (47). Although there is consensus that these metrics provide insight on protein quality, they do not adequately account for the impact of protein digestion and absorption kinetics (nutrikinetics) which play a pivotal role in protein metabolism. The most widely used example of this limitation is that whey protein and casein proteins have the same PDCAAS and both boast DIAAS greater than 1, but the rate of post-prandial amino acid appearance of casein is much slower. Studies have shown that faster appearing protein sources may be even more beneficial for muscle health during aging (48, 49). More research needs to be done to fully understand the impact of food processing and other macronutrients and micronutrients contained in plant-based proteins and food matrices on protein digestion including the kinetics of digestion and absorption. Additionally, given the variability in amino acid profiles among plant-based protein sources (24), improving their digestibility and nutrikinetics becomes paramount. Transparent information on protein quality via PDCAAS or DIAAS and additional information on nutrikinetics can help support a sustainable food system where nutrient dense food choices can be made from an informed perspective.

In conclusion, the integration of acid-activated proteases from the S53 family offers a promising avenue in the near term to enhance the digestibility and bioavailability of plant-based proteins to ensure there is minimal nutritional compromise when making sustainable dietary choices. In the longer-term, protein nourishment will be a centerpiece of precision nutrition. The issue of protein quality of plant-based diets becomes more pressing for populations of need including physically active adults and older adults that have greater protein needs and difficulty reaching recommended daily targets for protein intake, especially when consuming a vegan or vegetarian diet. As the world gravitates towards more sustainable dietary solutions, optimizing the nutritional value of these proteins is essential for both ecological balance and human health.

## Data availability statement

The original contributions presented in the study are included in the article/Supplementary material, further inquiries can be directed to the corresponding authors.

## Author contributions

WM: Conceptualization, Data curation, Formal analysis, Funding acquisition, Investigation, Methodology, Project administration, Supervision, Visualization, Writing – original draft, Writing – review & editing. CJ: Data curation, Formal analysis, Project administration, Validation, Visualization, Writing – original draft, Writing – review & editing. KM: Conceptualization, Data curation, Formal analysis, Methodology, Project administration, Writing – original draft, Writing – review & editing. EF: Writing – original draft, Writing – review & editing. JH: Writing – review & editing. JG: Writing – review & editing, Conceptualization. JS: Conceptualization, Funding acquisition, Investigation, Resources, Supervision, Writing – review & editing.
