# Supplementary material for: Acid-active proteases to optimize dietary protein digestibility: a step towards sustainable nutrition
Source: Front Nutr. 2024 Feb 8;11:1291685. doi: 10.3389/fnut.2024.1291685 (PMC10881760; doi:10.3389/fnut.2024.1291685)
Supplement: Supplementary file 1 [file Data_Sheet_1.docx]

Acid-Active Proteases and Their Role in Optimizing Dietary Protein Digestibility: A Step Towards Sustainable Nutrition

Wai Shun Mak^1,3*^, Chloe Jones^3^, Kevin McBride^3^, Emily AP Fritz^4^, Julie Hirsch^3^, J. Bruce German^2^, Justin B Siegel^1,3,5,6^

^1^Department of Chemistry, University of California Davis, Davis, California, USA

^2^Department of Food Science and Technology, University of California Davis, Davis, California, USA

^3^Digestiva, Inc Davis, California, USA

^4^Empowered Science, LLC South Barrington, Illinois, USA

^5^Genome Center, University of California Davis, Davis, California, United States of America

^6^Department of Biochemistry and Molecular Medicine, University of California Davis, Davis, California, United States of America

*** Correspondence:**

**Supplementary Information**

**Supplementary figure 1**. Qualitative analysis of protein digestibility on 31 protein sources by 12 proteases from the S53 family by SDS-PAGE analysis.

**Supplementary figure 2.** *Digestibility as percent degree of hydrolysis of protein sources when subjected to protease P24 using the INFOGEST 2.0 protocol. GP = Gastric Phase (120 minutes), IP 10 = Intestinal Phase at 10 minute timepoint, IP 120 = Intestinal Phase at 120 minute timepoint, Simulated digestive fluids are those prescribed by the INFOGEST 2.0 protocol as provided in the methods section. During the Gastric Phase (GP) simulated gastric fluid (SGF) was used, During the Intestinal Phase (IP) digesta from the gastric phase was added to simulated intestinal fluid (SIF) and analyzed at 10 minutes (IP 10) and 120 minutes (IP 120). Significance is noted with * at p<0.05.*

**Supplementary method**. Detail methods on sample preparation for protein digestibility screening on 31 protease sources.

**Supplementary table 1.** Uniprot accession code and species information for the 12 proteases tested in this work.

**Supplementary Table 1.** Protease activity proteins. All protein samples are classified according to the following: Plant: grains, legumes, nuts, seeds, protein extracts, and Animal origin

|  | - Classification | - Protein | - Source Detail |
| --- | --- | --- | --- |
| - 1 | - Grains | - Barley | - Signature Select brand |
| - 2 | - Grains | - Rye berry | - Organic Rye Berries, 28 oz (793 g) |
| - 3 | - Legumes | - Baby lima bean | - Camellia Brand, Dry Bean, 1lb Bag |
| - 4 | - Legumes | - Black bean | - Verde Valle brand |
| - 5 | - Legumes | - Chickpea | - Great Value garbanzo beans |
| - 6 | - Legumes | - Cowpea | - Organic Cowpea Red Whole (2 Lbs) |
| - 7 | - Legumes | - Fava bean | - Bob’s Red Mill brand |
| - 8 | - Legumes | - Lady cream bean | - Camellia Brand, Dry Bean (1 Pound Bag) |
| - 9 | - Legumes | - Lentil |  |
| - 10 | - Legumes | - Lupine bean | - Camellia Brand |
| - 11 | - Legumes | - Mung bean | - Red mung beans (Caravelle Adzuki beans) |
| - 12 | - Legumes | - Navy bean | - Camellia Brand, Dry Bean (1 pound) |
| - 13 | - Legumes | - Pea |  |
| - 14 | - Legumes | - Peanut | - C.H. trading company |
| - 15 | - Legumes | - Pink bean | - Camellia Brand, Dry Bean (1 Pound Bag) |
| - 16 | - Legumes | - Pinto bean | - El Mexicano brand |
| - 17 | - Nuts | - Almond | - Signature Select brand |
| - 18 | - Nuts | - Cashew | - Signature Select brand |
| - 19 | - Nuts | - Pistachio | - Signature Select brand |
| - 20 | - Seeds | - Buckwheat | - Arrowhead Mills buckwheat groats |
| - 21 | - Seeds | - Chia seed | - Carrington Farms brand |
| - 22 | - Seeds | - Quinoa | - Roland white quinoa |
| - 23 | - Protein extract | - Hemp protein concentrate | - Manitoba harvest Hemp pro50 |
| - 24 | - Protein extract | - Pea protein isolate | - NowSports Organic pea protein powder |
| - 25 | - Protein extract | - Soy protein isolate | - WAS soy protein powder |
| - 26 | - Animal | - Beef | - Ground beef |
| - 27 | - Animal | - Casein (milk protein) | - Gold Standard brand |
| - 28 | - Animal | - Chicken | - Tyson brand |
| - 29 | - Animal | - Pork | - Pork loin |
| - 30 | - Animal | - Salmon | - Walmart |
| - 31 | - Animal | - Turkey | - Jennie-O Ground turkey breast |

**Supplementary figure 1**. Qualitative analysis of protein digestibility on 31protein sources by 12 proteases from the S53 family by SDS-PAGE analysis.

**Supplementary Figure 2**


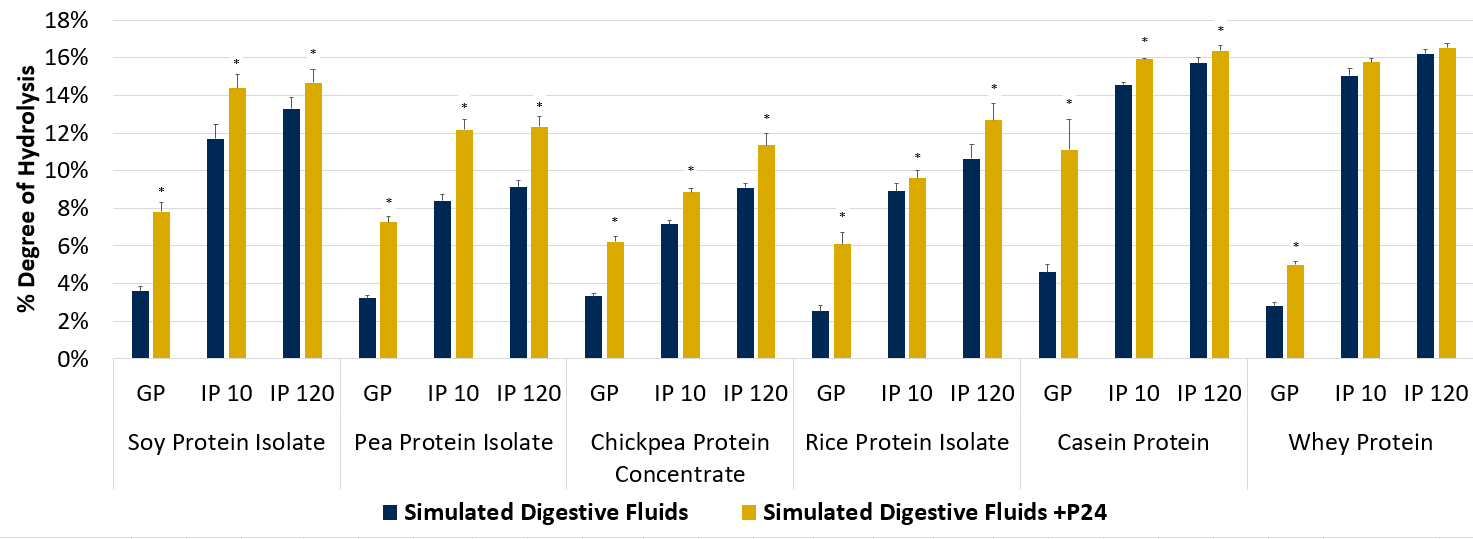

*Digestibility as percent degree of hydrolysis of protein sources when subjected to protease P24 using the INFOGEST 2.0 protocol. GP = Gastric Phase (120 minutes), IP 10 = Intestinal Phase at 10 minute timepoint, IP 120 = Intestinal Phase at 120 minute timepoint, Simulated digestive fluids are those prescribed by the INFOGEST 2.0 protocol as provided in the methods section. During the Gastric Phase (GP) simulated gastric fluid (SGF) was used, During the Intestinal Phase (IP) digesta from the gastric phase was added to simulated intestinal fluid (SIF) and analyzed at 10 minutes (IP 10) and 120 minutes (IP 120). Significance is noted with * at p<0.05.*

**Supplementary method**. Detail methods on sample preparation for protein digestibility screening on 31 protein sources.

For the optimization of proteolytic digestion assays, various protein sources were systematically processed for substrate preparation. The sources were categorized based on their initial form and composition, and separate methods were employed for their conversion into a uniform substrate powder suitable for downstream applications.

Animal-Derived Proteins

For protein sources derived from Beef, Turkey, Chicken, Pork, Casein, and Salmon:

1. Meat samples were initially processed by microwaving in a 1L borosilicate glass beaker for 10 minutes.
2. Post-microwaving, the samples were patted dry using laboratory-grade paper towels and subjected to mechanical disintegration using a commercial-grade blender in the presence of dry ice until a fine, frozen powder was obtained.
3. The resulting powder was aliquoted into 50 mL Falcon tubes and stored at -80°C until further use.
4. Prior to any analytical procedures, samples were conditioned in the freezer for at least 48 hours to ensure complete sublimation of any residual dry ice, thereby ensuring that the mass measurements were reflective solely of the protein content.

Legume-Derived Proteins

For protein sources from Mung beans, Pea, Pinto Beans, Black beans, Lentils, Chickpea, Lupine beans, Cowpea, Baby Lima, Pink Beans, Lady Cream Beans, and Navy Beans:

1. An aliquot of 100g of each legume was added to a 1L borosilicate glass beaker containing 500 mL of deionized water.
2. The mixture was heated on a laboratory hotplate at 55°C for 1.5 hours or until 95% of the water had evaporated.
3. After cooling to room temperature, the legumes were also blended with dry ice to obtain a fine powder.
4. The legume-derived powders were stored in 50 mL Falcon tubes at -80°C until required for assays.
5. As with animal-derived proteins, samples were preconditioned in the freezer for 48 hours prior to use.

Nut and Seed Proteins

For proteins derived from Almonds, Cashew, Pistachios, Peanut, Quinoa, Buckwheat, Chia seeds, and Hemp protein concentrate:

1. The seeds and nuts were mechanically disintegrated using a commercial-grade blender until a fine powder was obtained.
2. The resulting powders were aliquoted into 50 mL Falcon tubes and stored at room temperature until further use.

Protein Isolates

For proteins such as Pea protein isolate and Soy protein isolate:

1. The protein isolates were received in a pre-processed, powdered form and were directly aliquoted into 50 mL Falcon tubes without further processing.

All the processed protein sources were subjected to sieving through a laboratory-grade sieve to ensure homogeneity of particle size before storage. The protein concentrations for use in proteolytic digestion assays were determined based on nutritional label information, aiming for a uniform concentration of 5 mg/mL across all substrates.

**Supplementary table 1.** Uniprot accession code and species information for the 12 proteases tested in this work.

| **Uniprot Accession Number** | **Species** |
| --- | --- |
| A0A1Q4E140_9PSEU | [Pseudonocardia sp. 73-21](https://www.uniprot.org/taxonomy/1895809) |
| A0A1H3HWF1_9ACTN | [Modestobacter sp. DSM 44400](https://www.uniprot.org/taxonomy/1550230) |
| A0A0G3LJA6_XANCT | [Xanthomonas campestris pv. translucens](https://www.uniprot.org/taxonomy/343) |
| A0A0A6QII6_9BURK | [Paraburkholderia sacchari](https://www.uniprot.org/taxonomy/159450) |
| A0A0F0E4W8_9BURK | [Burkholderiaceae bacterium 26](https://www.uniprot.org/taxonomy/1619951) |
| A0A0G3EQQ7_9BURK | [Pandoraea thiooxydans](https://www.uniprot.org/taxonomy/445709) |
| A0A068NRV5_9BACT | [Fimbriimonas ginsengisoli Gsoil 348](https://www.uniprot.org/taxonomy/661478) |
| Q8RR56_9BACI | [Bacillus sp. MN-32](https://www.uniprot.org/taxonomy/198803) |
| PICP_PSESR | Pseudomonas sp. (strain 101) |
| A0A1C6LXN3_9BURK | [Variovorax sp. HW608](https://www.uniprot.org/taxonomy/1034889) |
| A0A1M7QZH1_9SPHI | [Mucilaginibacter sp. OK098](https://www.uniprot.org/taxonomy/1855297) |
| A0A1N6JUR9_9BRAD | [Bradyrhizobium erythrophlei](https://www.uniprot.org/taxonomy/1437360) |
